# Non-covalent functionalization of graphene sheets by pyrene-endcapped tetraphenylethene: Enhanced aggregation-induced emission effect and application in explosive detection

**DOI:** 10.3389/fchem.2022.970033

**Published:** 2022-08-11

**Authors:** Yumin Zhang, Huanran Li, Qing-Yun Wu, Lin Gu

**Affiliations:** School of Chemical Engineering and Technology, Sun Yat-sen University, Zhuhai, China

**Keywords:** functionalized graphene, aggregation-induced luminescence, 2,4-dinitrotoluene, tetraphenylethylene, pyrenol

## Abstract

In this work, a dispersible graphene-based material with a characteristic of aggregation-induced emission (AIE) was prepared by wet chemical reduction of graphene oxide (GO). During the GO reduction process, a conjugated molecule TPEP containing tetraphenylethylene (TPE) and pyrene was employed as a stabilizer because of the *π*–π interactions and the wrapping effect. The as-prepared rGO-TPEP not only has good dispersion in solution but also processes the AIE feature. Its fluorescence intensity is 2.23 times higher than that of TPEP at the same condition. The unique optical properties and AIE effect enable the rGO-TPEP as a chemical sensor for highly sensitive explosive detection in aggregated state and solid state. In the aggregated state, trace 2,4-dinitrotoluene (DNT) can be detected by the rGO-TPEP even when the concentration is as low as 0.91 ppm, and the quenching constant is as high as 2.47 × 104 M^-1^.

## Introduction

In recent years, graphene has become the research highlight due to its remarkable electrical (Chu et al., 2016; Tong et al., 2017; Fan et al., 2019), mechanical (Liu et al., 2021), thermal (Yang et al., 2019), (Liu et al., 2019), and optical properties (Li et al., 2020). Graphene is composed of single-layer carbon atoms, with a special structure of six-membered ring formed by sp^2^ hybridization and large delocalized *π* bond, which endows its stability and electronic mobility. However, the performance of graphene is dependent on its dispersion in practical application. The graphene is easy to aggregate due to the strong van der Waals force between the graphene layers (Sun et al., 2021), (Palermo et al., 2016). Therefore, the accessibility of the dispersible graphene is a prerequisite for most advanced applications.

Generally, chemical and non-covalent modifications are commonly used to prepare dispersible graphene. The former method employs specific chemically active functional groups to convert sp^2^-hybridized C=C bonds into sp^3^-hybridized C-C bonds (Xu et al., 2018), which would induce defects in graphene and further reduce its intrinsic electrical and thermal conductivity. In contrast, the non-covalent modification has little damage on the structure of graphene and can maintain most of the original properties (Choi et al., 2010; Lee et al., 2011; Gaire et al., 2021). In past decades, conjugated molecules were used as the dispersant of the graphene, such as 1-pyrenebutyrate (Xu et al., 2008), sulfonated polyaniline (Bai et al., 2009), dendronized perylene bisimides (Kozhemyakina et al., 2010), polyacetylenes (Xu et al., 2012a), and carboxylated oligoanilines (Gu et al., 2015). These dispersants could help graphene stay stable in solution via *π*–π interaction, van der Waals force, or electrostatic repulsion. The dispersant with conjugated structure usually has fluorescence properties, which however will be extensively quenched as the graphene is mixed due to the electron or energy transfer (Xu et al., 2008). Thus, non-covalent functionalized graphene with enhanced fluorescence emission would have promising applications in chemical sensors, optoelectronic devices, and biomedicine.

For the traditional fluorescence molecules, molecular aggregation caused by *π*–π stacking or hydrophobic interactions will result in the fluorescence quench, which is the reason of the quenching ability of graphene (Qi et al., 2012). In 2001, Tang’s team discovered a class of novel fluorescent molecules, AIEgens, which do not emit light in dilute solution but emit light in the aggregation state, called “aggregation-induced emission effect” (AIE effect) (Luo et al., 2001). However, in previous reports, the fluorescent was also easily quenched when AIE was used to chemically modify graphene, which limits the wide applications of graphene (Xu et al., 2012b; Zhang et al., 2018; Qin et al., 2019). In contrast, the fluorescence intensity is depended on the content of the non-covalent AIE-modified graphene, which can enhance the fluorescence of AIE molecules in a certain range (Qi et al., 2012). Li et al. used conjugated polymers containing AIE molecule, carbazole, and phenyl groups as stabilizers to prepare soluble graphene materials, whose AIE effect was significantly enhanced by graphene (Li et al., 2017).

In this communication, a conjugated molecule TPEP containing pyrene and tetraphenylethylene (TPE) was designed and synthesized, which was used as a stabilizer to prepare the non-covalent functionalized rGO via wet chemical reduction of graphene oxide (GO). The as-prepared rGO-TPEP not only had good dispersion in solution but also processed the AIE feature. Compared with TPEP, the AIE effect of rGO-TPEP was greatly enhanced. Furthermore, rGO-TPEP can be used for the detection of explosive as low as 0.91 ppm, and could be employed as a solid probe for the detection of DNT.

**FIGURE 1:**
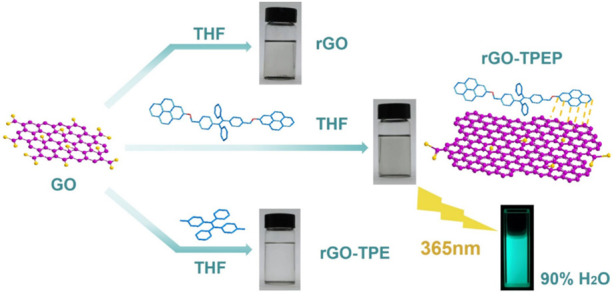
Schematic illustration of the preparation of the rGO-based materials.

As illustrated in Supplementary Figure S1 (Supporting Information), highly conjugated molecule TPEP containing pyrene and TPE was synthesized via McMurry coupling reaction, bromination, and substitution reaction, whose chemical structure was proved by 1H NMR (Supplementary Figure S2A, Supporting Information). TPEP was used as a stabilizer to functionalize rGO in THF, where GO was dispersed in THF via sonication for 0.5 h, then reduced to rGO by hydrazine hydrate in the presence of TPEP (Figure 1). After reduction, the solution color changed from brown to black, indicating the construction of sp^2^-conjugated structures. The as-prepared rGO-TPEP was stable in THF after removing the insoluble rGO *via* centrifugation. Compared with TPEP, rGO-TPE displayed a more transparent color, revealing less rGO in rGO-TPE than that in rGO-TPEP. Without conjugated molecules, rGO was only stable for several minutes, presenting its poor dispersion in THF solution. Therefore, TPEP is a better stabilizer for functionalized rGO due to the *π–*π interaction between rGO and TPEP.

FTIR was carried out to confirm the non-covalent functionalization of rGO, as shown in Supplementary Figure S2B (Supporting Information). For GO, several characteristic bands at 3,400–3,600, 1,730, and 1,620 cm^-1^ were ascribed to the hydroxyl, carbonyl groups, and C=C skeletal vibration, respectively (Acik et al., 2011). After modification, some characteristic peaks of TPEP at 2,926 and 2,855 cm^-1^ appeared for rGO-TPEP, which corresponded to the CH_2_ vibration. Furthermore, the peak at 1,600 cm^-1^ in TPEP belonged to the stretching vibration of the benzene ring, which was shifted to 1,660 cm^-1^ after modification, indicating the successful non-covalent functionalization of rGO.

**FIGURE 2:**
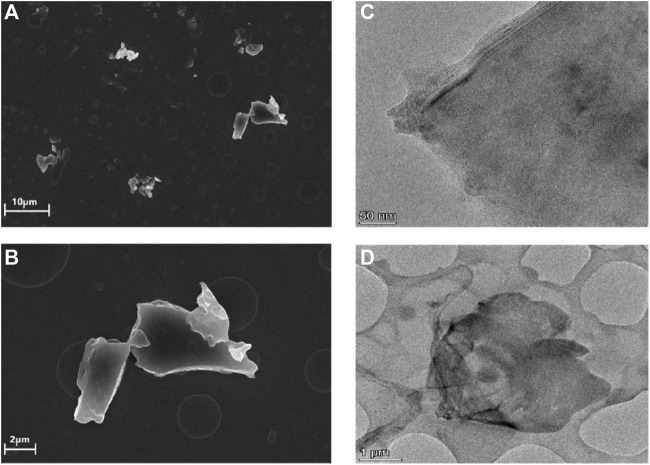
SEM **(A,B)** and TEM images **(C,D)** of rGO-TPEP.

The morphology of the obtained rGO-TPEP was observed by SEM and TEM (Figure 2). In the SEM image, the rGO-TPEP presented obvious flakes with a size of micron. In the TEM image, it can be seen clearly that the rGO-TPEP was ultrathin, which had some wrinkles and folded areas.

**FIGURE 3:**
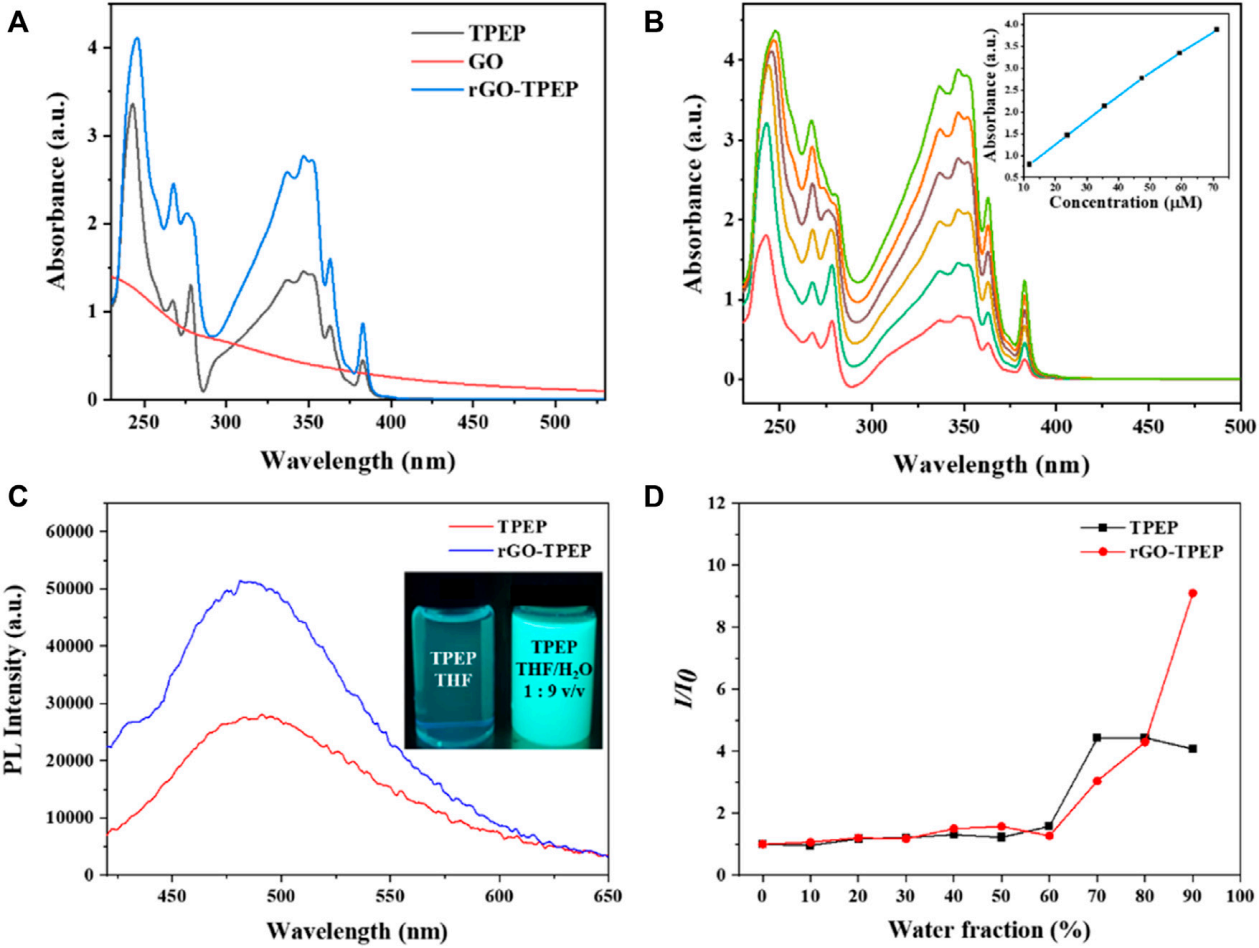
**(A)** UV-vis spectra of GO, TPEP, and rGO-TPEP in THF. **(B)** UV-vis spectra of rGO-TPEP at different concentrations, and inset shows concentration–absorption (at 347 nm) linear relationship. **(C)** Fluorescence spectra of TPEP and rGO-TPEP in THF/H_2_O mixed solution (water content 90%, λex = 350 nm). **(D)** PL peak intensity ratios of TPEP and rGO-TPEP at different water fractions in THF/H_2_O mixtures.

The absorption spectra of rGO-TPEP and TPEP in THF were measured via UV-vis spectroscopy and displayed in Figure 3A. GO showed an absorption band at 228 nm and a shoulder peak at 300 nm, which was attributed to the *π*–*π* * transition of C=C bond and the n–*π* * transition of C=O bond, respectively (Li et al., 2017). After reduction, rGO aggregated in THF, which made its UV-vis spectrum difficult to be measured. However, rGO-TPEP and TPEP showed two strong absorption peaks at 245 and 347 nm, corresponding to the *π*–*π* * transition of C=C bonds in the benzene ring and pyrene unit of TPEP, respectively. The peak intensity of the rGO-TPEP ascribing to the pyrene unit increased along with a blue shift, demonstrating the π−π interaction between rGO and TPEP. To confirm the good solubility of rGO-TPEP, the absorption spectra of different concentrations of rGO-TPEP were measured, as shown in Figure 3B. The absorbance of rGO-TPEP increased with the increase of concentration, in which the solution exhibited Lambert–Beer behavior. There is a linear relationship between the absorbance at 347 nm and the concentration at 11.88–71.28 μg/ml, which showed that the rGO-TPEP had good solubility in THF.

As is well known, the TPE unit in TPEP is a typical AIE structure, which is non-fluorescent in solution but is highly emitting in the aggregated state. It has been reported that the conjugated molecules containing TPE still have AIE activity (Tu et al., 2016). Therefore, the photoluminescence (PL) properties of the synthesized TPEP were studied, as depicted in Figures 3C and D. In the inset photo of Figure 3C, the THF solution of TPEP presented a faint blue fluorescence under the irradiation of 365-nm UV lamp. Nevertheless, TPEP aggregated in THF/H_2_O mixture with a water fraction of 90 vol%, showing strong blue light and a single emission peak at 480 nm (Figure 3C). As seen in Figure 3D, the fluorescence of TPEP gradually increased with the increase of water content in the mixture. When the water content was less than 60 vol%, the fluorescence intensity was very weak, but then it increased rapidly. The fluorescence intensity increased by 4.07 times when the water content reached 90 vol%. This is a typical AIE effect. With the content of water increased, TPEP was gradually aggregated since TPEP was insoluble in water, and TPEP emitted bright blue fluorescence. However, when the water content reached to 70 vol%, it was close to the intensity of 90 vol% by both effect of AIE and the quenching of pyrene.

In addition, the AIE characteristics of rGO-TPEP were investigated using the same method. Interestingly, the shape and the shift of the peak did not change after combined with rGO, but the AIE effect was greatly enhanced, which is different from the previous results of graphene quenching the fluorescence of organic dyes (Xu et al., 2008; Xu et al., 2012a; Tu et al., 2016). When the water content exceeded 70 vol%, the fluorescence intensity of rGO-TPEP changed faster than TPEP, while the water content of the mixture reached 90 vol%. The PL intensity of rGO-TPEP changed by 9.09 times, which is 2.23 times than that of TPEP. As can be seen from the picture in Figure 3C, rGO-TPEP has higher emissivity under the same conditions when compared with TPEP. This may be due to the hydrophobicity of reduced graphene oxide, and the nanosheets tend to aggregate when added water. Thus, rGO wrapped by TPEP restricted the rotation of propeller-like phenyl rings further, leading to high fluorescence intensity. Although it has been reported that the rGO has fluorescence quenching effect on fluorescent molecule with the difference of the distance as well as concentration (Qi et al., 2012; Tan et al., 2014), the content of rGO may be not enough to quench the fluorescence of TPEP molecule, and large amount of TPEP probably attached on both sides of rGO nanosheets.

Explosives are usually a kind of electron-deficient nitro organic compounds, including 2,4,6-trinitrotoluene (TNT), 2,4-dinitrotoluene (DNT), and picric acid (PA). They not only pose a threat to the security of the country and society but also pose serious health hazards to human body. The explosive sensor based on photoluminescence (PL) has attracted extensive attention because of its high sensitivity. Explosives can cause fluorescence quenching of electron-rich probes through the photoinduced electron transfer (PET) mechanism (Feng et al.,2014). However, there is an aggregation-caused quenching (ACQ) phenomenon in traditional fluorescence molecules, that is, the fluorescence will weaken or even not emit light at high concentration, which greatly limits the accuracy and concentration of fluorescent molecular probes. The molecules with AIE effect have excellent sensitivity in detecting explosives. In recent years, there are many reports on the use of AIE molecules in explosive detection (Li et al., 2016; Dong et al., 2018; Zhu et al., 2022). Electron-rich TPE groups and electron-deficient nitro organic compounds can show a very sensitive fluorescence response through PET, so they can detect explosives with high sensitivity (Namgung et al., 2018). TPEP is a typical AIE molecule, and when it is non-covalently functionalized with rGO, its AIE effect is significantly enhanced. In view of the special function of luminescent rGO-TPEP, it can be used for the detection of explosives. We found that it showed high sensitivity to 2,4-dinitrotoluene (DNT). The detection process could be carried out in both aggregation state and solid state with high sensitivity, which made the luminescent rGO-TPEP suitable for tracking and detecting DNT. When rGO-TPEP with AIE activity is used as a probe, the existence of rGO can adsorb electron-deficient nitro organic compounds by *π*–π and electrostatic interactions, which can further improve its sensitivity to DNT.

**FIGURE 4:**
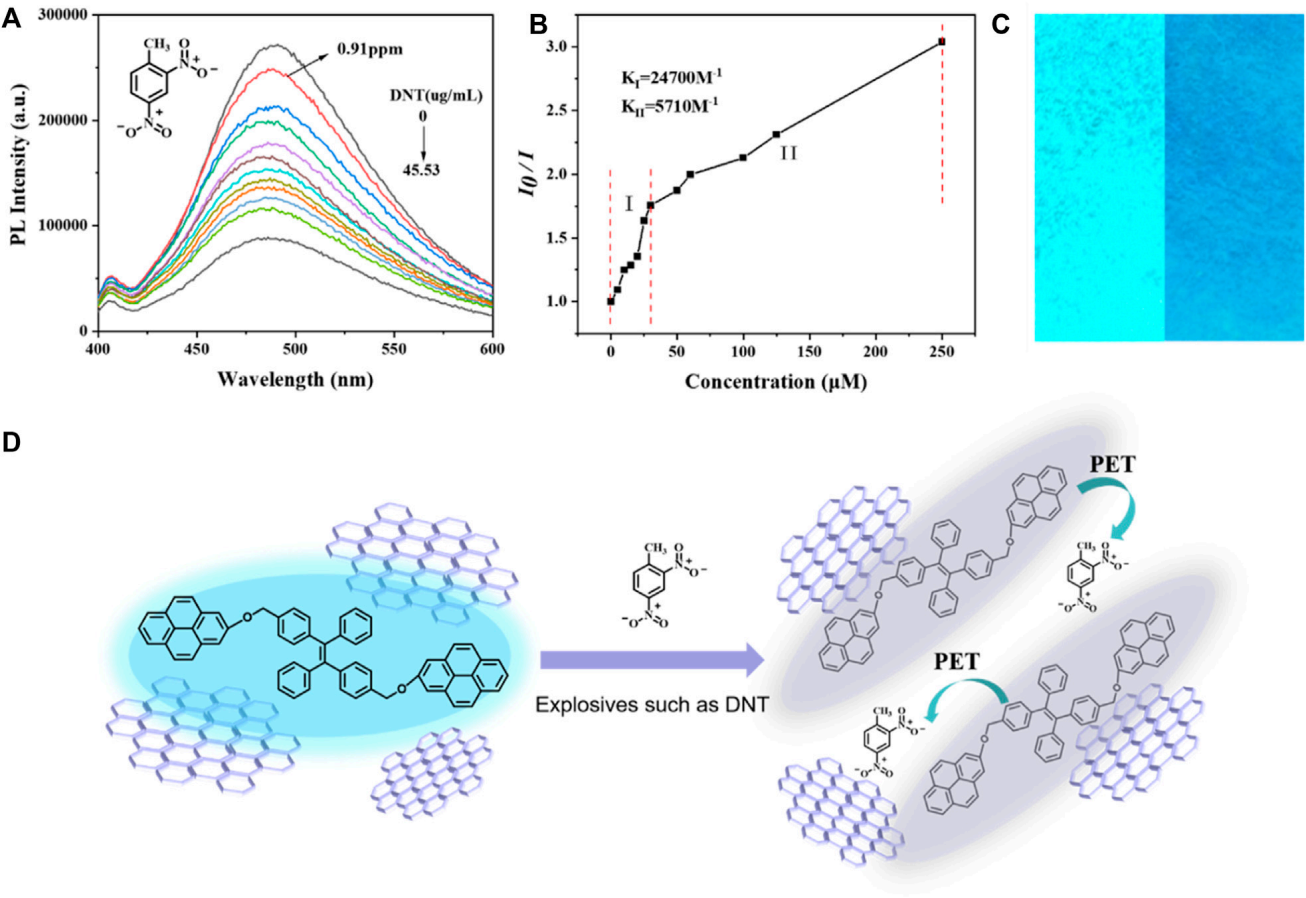
**(A)** PL spectra of rGO-TPEP with different DNT amounts in a THF/H_2_O mixture with a 99% water fraction. rGO-TPEP concentration: 10^–5^ M and excitation wavelength: 350 nm. **(B)** Relative PL diagram under different DNT concentrations (I0 = intensity at [DNT] = 0 μM). **(C)** Fluorescence images of rGO-TPEP adsorbed on filter papers dipped into a THF/H_2_O mixture (1:99, v/v, left) and then dipped into a DNT solution (right). rGO-TPEP concentration: 10^–4^ M and DNT concentration: 0.25 mM. **(D)** Mechanism of explosive DNT detection.

Fluorescence quenching could be detected even when the DNT concentration was as low as 5 µM (0.91 ppm), and the degree of quenching increased with the increase of DNT concentration (Figure 4A). In the linear graph of relative PL intensity (I_0_/I) and DNT concentration (Figure 4B), when DNT was lower than 30 µm (5.46 ppm), the correlation coefficient (R^2^) was 0.95315, and the quenching constant was 2.47 × 10^4^ M^-1^. In the concentration range of 50–250 µM (9.1–45.53 ppm), the quenching constant was 5.71 × 10^3^ M^-1^. The higher value of quenching constant in the lower concentration means higher sensitivity and accuracy of detection for a small amount of explosives, so rGO-TPEP is more suitable for the detection of trace DNT.

In addition to the aggregated solution state, it can also be detected in the solid state, which is even more convenient. The filter paper was dipped in the rGO-TPEP solution and dried to get the test paper. When the paper was dipped in explosive solution, it could be seen that the luminescence of the filter paper (right picture) was significantly weakened, while the fluorescence effect of the filter paper not soaked by DNT was very strong. This confirms the prepared rGO-TPEP could also be used as a solid probe in real application. It can be seen that the luminescent graphene has a good application prospect in explosion detection.

In conclusion, non-covalent functionalized graphene was designed and prepared based on the π−π interaction between graphene and TPEP via the wet chemical reduction of GO. The as-prepared rGO-TPEP not only had good dispersion in solution but also processed the AIE feature. Compared with TPEP, the AIE effect of rGO-TPEP was greatly enhanced. Furthermore, rGO-TPEP could be used for the detection of explosive as low as 0.91 ppm and could be employed as a solid probe for the detection of DNT. These results offer new opportunities for developing new graphene-based materials with promising applications in chemical sensors, optoelectronic devices, and biomedicine.

## Data Availability

The original contributions presented in the study are included in the article/Supplementary Material; further inquiries can be directed to the corresponding authors.
