# Supplementary material for: Non-covalent functionalization of graphene sheets by pyrene-endcapped tetraphenylethene: Enhanced aggregation-induced emission effect and application in explosive detection
Source: Front Chem. 2022 Aug 11;10:970033. doi: 10.3389/fchem.2022.970033 (PMC9403001; doi:10.3389/fchem.2022.970033)
Supplement: Supplementary file 1 [file DataSheet1.docx]

**Supporting Information**

**Non-covalent functionalization of graphene sheets by pyrene-endcapped tetraphenylethene: Enhanced aggregation-induced emission effect and application in explosive detection**

Yumin Zhang^1^, Huanran Li^1^, Qing-Yun Wu^1*^, Lin Gu^1^**^^[[1]](#footnote-0)^*^**

^1^ School of Chemical Engineering and Technology, Sun Yat-sen University, Zhuhai 519082, China

**1. Experimental section**

**1.1** **Materials**

4-Methylbenzophenone, tetrahydrofuran (THF), dichloromethane (DCM), sodium sulphate (Na_2_SO_4_), potassium carbonate (K_2_CO_3_), *n*-hexane, *N*-bromosuccinimide (NBS) , dibenzoyl peroxide (BPO) , carbon tetrachloride (CCl_4_) , and petroleum ether were purchased from Shanghai Macklin Biochemical Co., Ltd. Zinc powder, 1-pyrenol, titanium tetrachloride (TiCl_4_), and hydrazine hydrate were purchased from Shanghai Aladdin Biochemical Technology Co., Ltd. Hydrochloric acid (HCl) was purchased from Xilong Scientific Co., Ltd. Graphene oxide (GO) was provided by Wuxi qihao environmental-friendly new material research Co., Ltd. Trichloromethane (CHCl_3_) was purchased from Guangzhou chemical reagent Co., Ltd.

**1.2** **Characterizations**

^1^H NMR spectra were recorded on a 400M Bruker AVANCE NEO spectrometer. Fourier transform infrared (FTIR) spectra were measured on a Spectrum100SystemB FTIR spectrophotometer in the range of 500–4000 cm^-1^. UV-vis spectra were obtained on a Shimadzu UV2600 UV spectrophotometer. Photoluminescence (PL) spectra were performed on a Edinburgh FS5 fluorescence spectrometer. Scanning electron microscopy (SEM) was carried out on a Zeiss Sigma 300. Transmission electron microscopy (TEM) was obtained on a FEI Talos F200s.

**1.3** **Synthesis of pyrene- endcapped tetraphenylethene (TPEP)**

**1.3.1** **Synthesis of 1,2-bis (4-methylphenyl) - 1,2-** **diphenylethene (TPE)**

[1,2-bis (4-methylphenyl)-1,2-diphenylethylene] was synthesized via McMurry coupling reaction according to the literature.^1^ The specific process is as follows: 5.89 g (30 mmol) of 4-methylbenzophenone, 5.88 g of zinc powder (90 mmol) and 100 mL of tetrahydrofuran were added in argon atmosphere. Place the flask in an ethanol bath at - 80 ℃ with magnetic stirring. 7.56 g (90 mmol) of titanium tetrachloride was added dropwise, the mixture was cooled to room temperature after reaction. The mixture was poured into dilute hydrochloric acid (HCl, 1M), extracted with dichloromethane to obtain an organic mixed phase. After removing water with anhydrous sodium sulfate, it was concentrated under reduced pressure. Finally, the mixture was purified with eluent (*V* *_hexane_*: *V* *_chloroform_* = 3:1) in silica gel column, and dried to obtain high-purity white solid product. The estimated yield is 91% (4.94 g).^1^ ^1^H NMR (400 MHz, CDCl_3_) *δ* (TMS, ppm): 7.15–6.98 (m, 10 H), 6.94–6.85 (m, 8 H), 2.26 (s, 6 H).

**1.3.2** **Bromination of 1,2-bis (4-methylphenyl) - 1,2-diphenylethene (TPE-Br)**

The specific process is as follows: 1.44g (4.0 mmol) [1,2-bis (4-methylphenyl)-1,2-diphenylethylene], 1.57g (8.8 mmol) of freshly recrystallized NBS, 0.01 g BPO and 60 mL of CCl_4_ were added and refluxed for more than 10 h, and gradually cooled to room temperature. The mixed solution was filtered, and the filtrate was concentrated under reduced pressure to obtain the crude product, which was purified with eluent (*V* *_hexane_*: *V* *_chloroform_* = 4:1) through silica gel column to obtain white solid product. The estimated yield is 56% (1.16 g). ^1^H NMR (400 MHz, CDCl_3_) *δ* (TMS, ppm): 7.18–7.06 (m,10 H),7.04–6.94 (m, 8 H), 4.41 (s, 4 H).

**1.3.3** **Synthesis of pyrene- endcapped tetraphenylethene (TPEP)**

The specific steps are as follows: 1-pyrenol (0.873 g, 4 mmol), potassium carbonate (0.553g, 4mmol), TPE-Br (1.08g, 2mmol) and tetrahydrofuran (THF, 40 ml) are added, and the mixture is refluxed overnight. Then the mixture was cooled to room temperature and concentrated to obtain the crude product, and then eluted and purified in a silica gel column with eluent (*V* *_chloroform_*: *V* _petroleum ether_ = 1:5) to obtain a white solid (1.2672g, 80% yield). ^1^H NMR (400 MHz, CDCl_3_) *δ* (TMS, ppm): 8.55–7.27(m,18H), 7.19–6.92 (m, 16H), 5.30 (s, 2H).

**1.4** **Synthesis of luminescent non-covalent functionalized graphene (****rGO-TPEP)**

Specific steps: TPEP (0.1402 g, 0.177mmol) was dissolved in THF (16.7 mL). Take 1 wt% graphene oxide (GO) aqueous solution, evaporate the water and dry it overnight, then grind it with a ball mill for 2 hours. The dried and ground GO (10 mg) was dispersed in THF (20 mL), and subjected to ultrasonic action at room temperature for 30 min, which was added dropwise to the TPEP solution in an argon atmosphere. After stirring for 2 h, added hydrazine hydrate (0.25 mL) and refluxed the mixture for 24 h, then it was cooled to room temperature and sonicated for 20min. To remove the precipitate, the mixture was centrifuged at 3000 rpm for 20 min and a stable black supernatant was collected. The solvent in the supernatant was removed to obtain a black solid (rGO-TPEP). Dry overnight under vacuum at 45 °C.


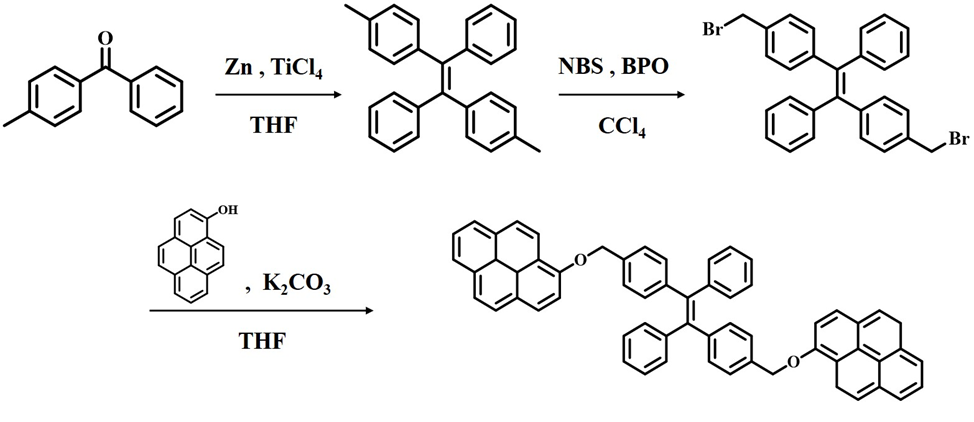


**Figure S1.** Synthetic route of TPEP.


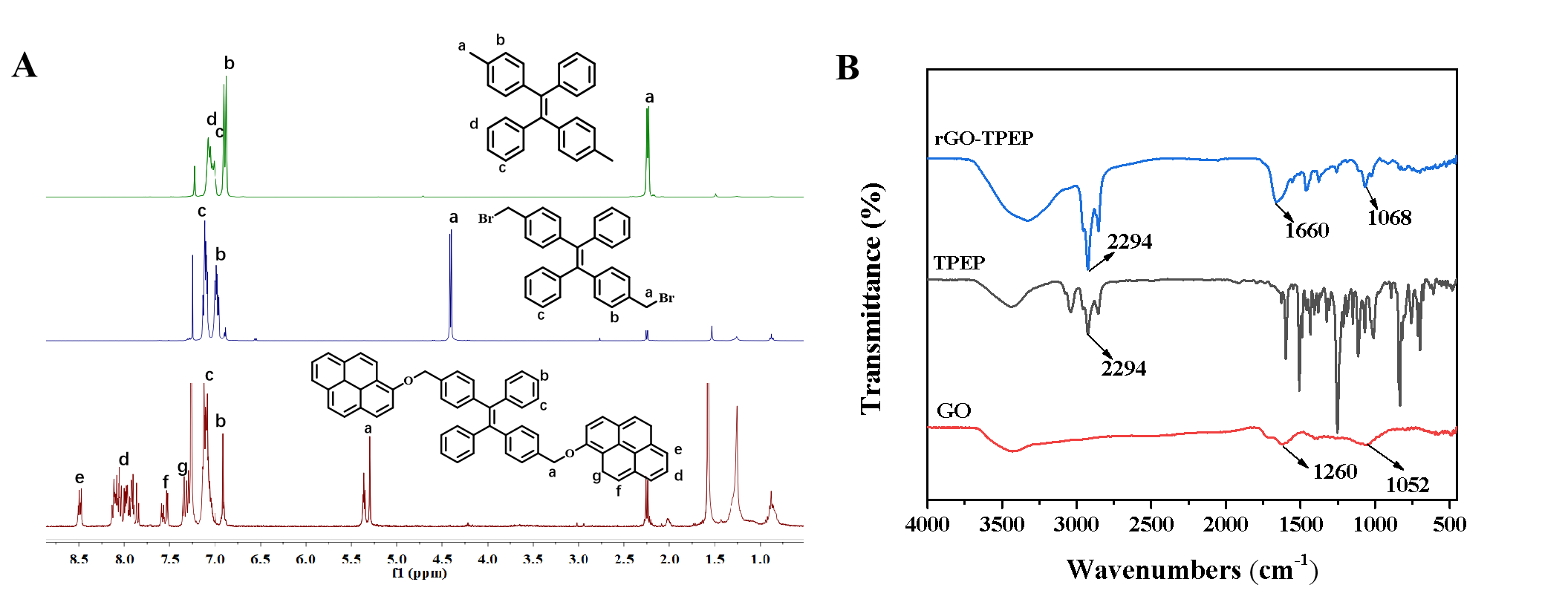


**Figure S2**. **(A)** ^1^H NMR spectra of TPE, TPE-Br and TPEP.

**(B)** FTIR spectra of GO, TPEP and rGO-TPEP.

**References**

1. Liang, J.; Shi, H.; Kwok, R. T. K.; Gao, M.; Yuan, Y.; Zhang, W.; Tang, B. Z.; Liu, B., Distinct optical and kinetic responses from E/Z isomers of caspase probes with aggregation-induced emission characteristics. *J Mater Chem B* **2014,** *2* (27), 4363-4370.

1. *Corresponding authors: E-mail: [gulin5@mail.sysu.edu.cn](mailto:gulin5@mail.sysu.edu.cn) (L. Gu); wuqingy5@mail.sysu.edu.cn (Q-Y Wu) [↑](#footnote-ref-0)
